# Resolution of Treatment-Refractory Prurigo Nodularis With Dupilumab: A Case Series

**DOI:** 10.7759/cureus.8737

**Published:** 2020-06-21

**Authors:** Jill K Wieser, Mary Gail Mercurio, Kathryn Somers

**Affiliations:** 1 Dermatology, University of Rochester Medical Center, Rochester, USA

**Keywords:** prurigo nodularis, dupilumab, pruritus

## Abstract

Prurigo nodularis is a pruritic skin condition that can present therapeutic challenges. We present a series of three patients diagnosed with prurigo nodularis who had failed several commonly trialed therapies, but experienced relief from symptoms and improvement in skin lesions following initiation of dupilumab therapy. All patients in this series lacked a diagnosis of atopic dermatitis and had lesions on the lower extremities, although other locations such as the trunk were also involved. Continued study of dupilumab in patients with prurigo nodularis is advocated.

## Introduction

Prurigo nodularis (PN) is a chronic cutaneous condition associated with intense pruritus. Treatment can be challenging as there are currently no FDA-approved therapies for PN. Topical and intralesional steroids are often trialed with varying success. Several systemic medications have been studied, such as thalidomide or methotrexate, but these can be associated with significant side effects [1]. Dupilumab, a fully humanized IL-4Rα antibody, has a favorable safety profile and is FDA-approved for the treatment of atopic dermatitis (AD), asthma, and chronic rhinosinusitis with nasal polyposis [2,3]. This drug has recently been demonstrated to be effective in the treatment of PN in the setting of an atopic history [4]. We present a case series of three patients without AD who experienced dramatic improvement in both itch and clinical appearance of lesions following treatment with dupilumab. All patients were treated with 600 mg loading dose subcutaneously (SQ) followed by 300 mg SQ every two weeks.

## Case presentation

Patient 1

**Figure 1:**
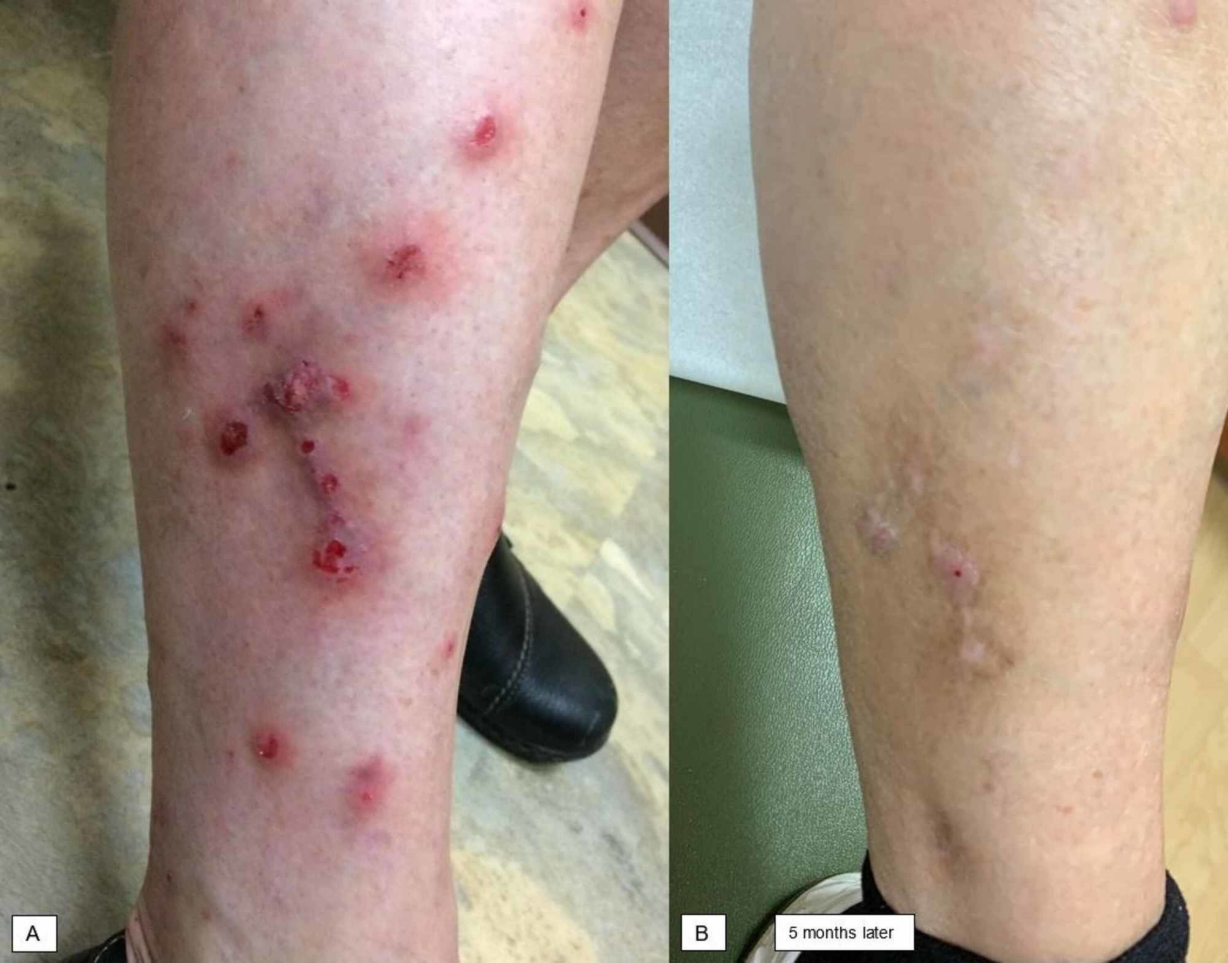
Patient 1 before and after initiation of dupilumab therapy. (A) Excoriated papules and nodules were present on the lower extremities. (B) Nearly complete clearance was noted five months after initiation of dupilumab.

A 66-year-old woman presented for PN affecting her torso and extremities (Figure 1A) that had been present for over two years. She had no history of atopic disease (i.e. asthma, allergic rhinitis, or AD). Two 4-mm punch biopsies were performed, one on the arm and the other on the chest, which were read as superficially traumatized spongiotic dermatitis with superficial dermal perivascular infiltrate including numerous eosinophils. She was treated with high-potency topical steroids, antihistamines, prednisone, methotrexate titrated to 25 mg daily, gabapentin titrated to 1,200 mg daily, N-acetyl cysteine, and oral antibiotics without resolution. She experienced side effects from the methotrexate including gastrointestinal upset, which was intolerable for her. Dupilumab was initiated, and significant improvement was noted at her follow-up appointment two months later; after five months of treatment, she was nearly clear (Figure 1B). She noted reduction in pruritus and denied side effects from dupilumab.

Patient 2

**Figure 2:**
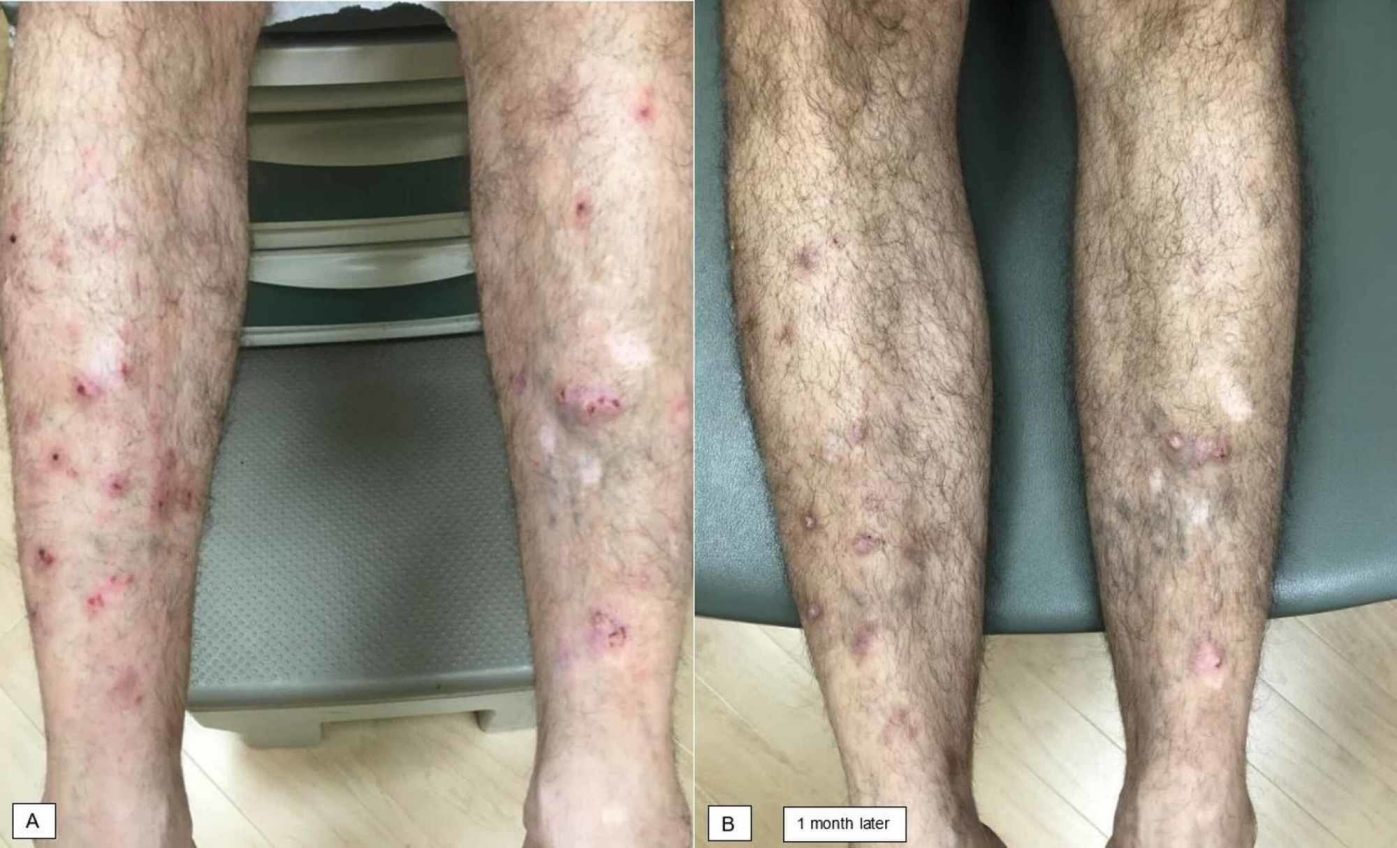
Patient 2 before and after initiation of dupilumab therapy. (A) Excoriated papules and nodules were noted on the lower extremities. (B) Significant improvement was noted one month after dupilumab initiation.

A 65-year-old man presented for PN on the lower extremities, groin, and trunk (Figure 2A). This had been previously treated with phototherapy and thalidomide, and had largely resolved until a few months prior to this visit. He had no history of atopy. Biopsy was deferred as nodules were clinically consistent with PN. He was treated with intralesional triamcinolone 10 mg/mL, topical triamcinolone 0.1% ointment, and gabapentin titrated to 600 mg daily. He did not experience significant relief with this regimen; therefore, dupilumab was initiated. He experienced a significant improvement in both pruritus and cutaneous lesions by his follow-up appointment one month later (Figure 2B). He noted a reduction in itch and denied adverse events from dupilumab.

Patient 3

**Figure 3:**
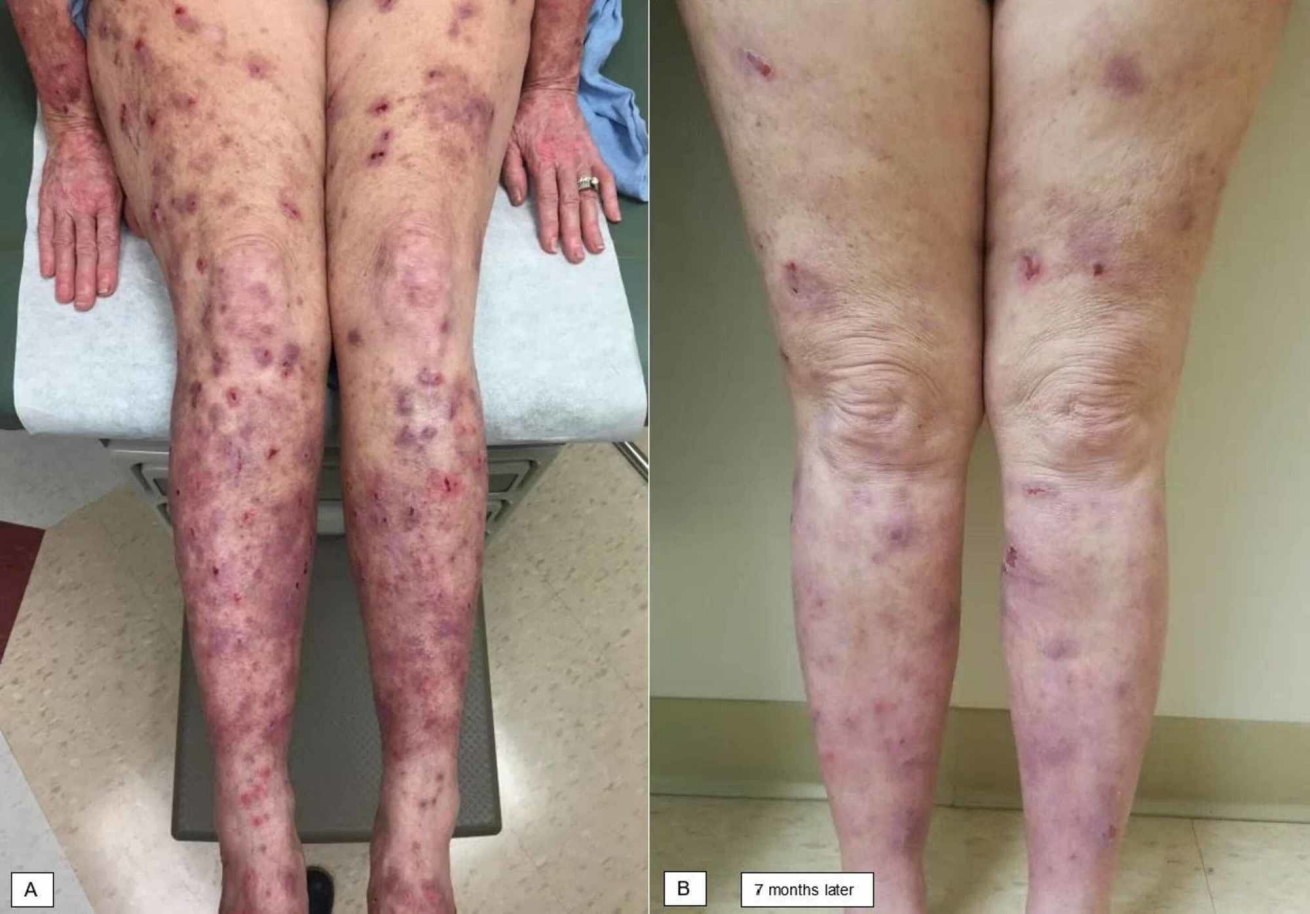
Patient 3 before and after initiation of dupilumab therapy. (A) Erythematous plaques and excoriated nodules were most prominent on the upper and lower extremities. (B) Marked improvement was noted seven months after starting dupilumab.

A 65-year-old woman presented with a three-year history of PN on the trunk and extremities (Figure 3A). She had a remote history of asthma and allergic rhinitis and no longer received treatment for these conditions, but no history of AD. Her PN was treated with high-potency topical steroids, mupirocin, and antihistamines without relief. Punch biopsies of nodules on the anterior legs described denuded epidermis with superficial to deep dermal chronic inflammation and fibrosis, consistent with PN. Gabapentin titrated to 2,700 mg daily, tacrolimus 0.1% ointment, triamcinolone 0.1% ointment, intralesional triamcinolone 10 mg/mL, and hydroxyzine 50 mg nightly did not sufficiently control pruritus; titration of the gabapentin and hydroxyzine was limited by fatigue. Dupilumab was started, and her symptoms and cutaneous nodules had significantly improved by her subsequent visit seven months later (Figure 3B). She endorsed a reduction in pruritus and denied side effects from dupilumab.

## Discussion

Chronic itch can have a significant impact on a patient’s quality of life, and strongly correlates with measures of anxiety and depression in patients with PN [5,6]. Effective therapeutic options are limited, and long-term treatment with off-label systemic medications, such as cyclosporine, methotrexate, or thalidomide, can result in significant side effects [1]. Dupilumab, which inhibits signaling of IL-4 and IL-13, has demonstrated efficacy and safety in a retrospective review of 90 AD patients with the generalized PN phenotype [4]. Recent evidence suggests that IL-4Rα activation enhances the responsiveness of sensory neurons to several pruritogens, and this may be the mechanism by which dupilumab reduces itch in AD and other pruritic diseases [7]. Expression of the T-helper type 2 cytokines (IL-4 and IL-13) has been observed in PN biopsies, suggesting that these mediators are important in development or perpetuation of this condition [8]. For these reasons, we speculated that dupilumab may be an effective symptomatic treatment for our patients. Although our patients experienced limited symptomatic relief from other systemic therapies, they all noted a significant reduction in pruritus when treated with dupilumab and did not report adverse events from this medication.

It is thought that up to 50% of PN cases occur in individuals with an atopic phenotype [9]. All three patients in this series have never been diagnosed with AD. However, dupilumab was highly effective clinically and symptomatically in all three patients. This adds to the growing literature on dupilumab effectively treating PN [10-12]. Although our figures are primarily of the lower extremities, equally efficacious improvement was noted in all involved areas in each of our patients. Based on our patients’ successes and those described in previous reports, we feel further research is warranted to further evaluate the long-term safety and efficacy of dupilumab in patients with PN with and without a history of AD. Research interest regarding PN appears to be increasing. Currently, a clinical trial is enrolling to evaluate the use of dupilumab in patients with PN who are not adequately controlled by topical therapies [13]. Additionally, a recent phase 2 trial involving nemolizumab, which inhibits IL-31 receptor A, suggested efficacy over placebo but was associated with gastrointestinal and musculoskeletal adverse events [14].

## Conclusions

Our case series highlights how quickly the benefits of dupilumab may be appreciated in PN patients without AD as demonstrated by relief of intractable pruritus and remarkable improvement in lesions. The side-effect profile of dupilumab may be preferable to other off-label therapeutic options at this time. Improvement in chronic pruritus has the potential to improve patients’ quality of life. Continued study of dupilumab in patients with PN is warranted.
